# Evaluation of the Antibacterial and Antibiofilm Effects of Ethyl Acetate Root Extracts from *Vernonia adoensis* (Asteraceae) against *Pseudomonas aeruginosa*

**DOI:** 10.1155/2023/5782656

**Published:** 2023-06-06

**Authors:** Mercy Masuku, Winnie Mozirandi, Stanley Mukanganyama

**Affiliations:** Department of Biotechnology and Biochemistry, University of Zimbabwe, Mt. Pleasant, Harare, Zimbabwe

## Abstract

There is an increase in mortality and morbidity in the health facilities due to nosocomial infections caused by multidrug-resistant nosocomial bacteria; hence, there is a need for new antibacterial agents. *Vernonia adoensis* has been found to possess medicinal value. Plant phytochemicals may have antimicrobial activity against some resistant pathogens. The antibacterial efficacy of root extracts against *Staphylococcus aureus* and *Pseudomonas aeruginosa* was investigated using the microbroth dilution method. All extracts from the roots had an inhibitory effect on the growth of both bacteria, with the most susceptible being *P. aeruginosa*. The most potent extract was the ethyl acetate extract which caused a percentage inhibition of 86% against *P. aeruginosa*. The toxicity of the extract was determined on sheep erythrocytes, and its effect on membrane integrity was determined by quantifying the amount of protein and nucleic acid leakage from the bacteria. The lowest concentration of extract used, which was 100 *µ*g/ml, did not cause haemolysis of the erythrocytes, while at 1 mg/ml of the extract, 21% haemolysis was observed. The ethyl acetate extract caused membrane impairment of *P. aeruginosa,* leading to protein leakage. The effect of the extract on the biofilms of *P. aeruginosa* was determined in 96-microwell plates using crystal violet. In the concentration range of 0–100 *µ*g/ml, the extract inhibited the formation of biofilms and decreased the attachment efficiency. The phytochemical constituents of the extract were determined using gas chromatography-mass spectrometry. Results of analysis showed the presence of 3-methylene-15-methoxy pentadecanol, 2-acetyl-6-(t-butyl)-4-methylphenol, 2-(2,2,3,3-tetrafluoropropanoyl) cyclohexane-1,4-dione, E,E,Z-1,3,12-nonadecatriene-5,14-diol, and stigmasta-5,22-dien-3-ol. Fractionation and purification will elucidate the potential antimicrobial compounds which are present in the roots of *V. adoensis*.

## 1. Introduction

Microbial infections are on the rise resulting in increased morbidity and motility in the healthcare settings [1]. Morbidity and mortality rates in patients suffering from nosocomial infections are attributed to the fact that the bacteria which cause the infections have developed mechanisms to resist most of the currently available antibiotics [2]. *Pseudomonas aeruginosa (P. aeruginosa)* and *Staphylococcus aureus (S. aureus)* are nosocomial bacteria that are mainly isolated from patients suffering from nosocomial infections [3]. Life threatening infections such as meningitis, pneumonia, and sepsis will occur as a result of infection by *S. aureus* [4]. Some strains of *S. aureus* have been shown to be resistant to a number of antibiotics including methicillin, tetracyclines, chloramphenicol, and aminoglycosides [5]. An increase in nosocomial infections caused by *S. aureus* is reported each year [6], due to a rise in antibiotic resistance of the bacteria [7]. Due to these limitations of the antibiotics, there is an increasing need for the development of antimicrobial agents that efficiently control and manage bacterial infectious diseases [8].

*P. aeruginosa* causes nosocomial infections such as urinary tract infections, healthcare-associated pneumonia [9], bloodstream infections, surgical site infections, and skin infections on burnt wounds [10]. Infections of *P. aeruginosa* are of major concern because the bacteria have become resistant to current antibiotics including *β*-lactams, macrolides, tetracyclines, co-trimoxazole, and some fluoroquinolones [7]. Resistance in *P. aeruginosa* is attributed to the ability of the bacteria to form biofilms [11, 12]. The ability of pathogens to resist antibiotics is significantly enhanced once they form biofilms [13] and antibiotics have been found to be less effective in biofilm-growing bacteria [14]. There is increasing evidence that biofilm-mediated infection facilitates the development of acute infectious diseases and recurrent infections [15, 16]. Novel strategies are, therefore, required to deal with these biofilm-mediated problems [17].

Plants have been used in the treatment of diseases since time immemorial, and some have been found to be important sources of antimicrobial agents [18]. One prominent plant which is commonly used in African ethnomedicine is *Vernonia adoensis (V. adoensis)*. In Tanzania, this plant has been traditionally used in African ethnomedicine for the treatment of fever and upper respiratory tract infections [19].

Another ethnomedicinal use of the leaves of the plant includes the treatment of malaria symptoms in Western Uganda [20]. The plant has been traditionally used in Kenya for the treatment of symptoms of sexually transmitted diseases such as gonorrhea [21].

Studies have shown that different parts of the plant have antimicrobial activity [22, 23]. Extracts from *V. adoensis* were found to possess some important pharmacological phytochemicals [24]. The aim of this study was to evaluate the antimicrobial activity of extracts from the roots of *V. adoensis, against S. aureus, and P. aeruginosa* and to analyse the bioactive compounds in the most potent extract of the plant using GC-MS analysis. Knowledge of the chemical constituents of plants is desirable as these may serve as new sources of compounds that can lead to the development of new antibacterial agents [25]. The study also aimed to determine the antibiofilm potential of the potent extract against the most susceptible bacteria.

## 2. Materials and Methods

### 2.1. Plant Material and Chemicals

*V. adoensis* plant was collected from Centenary (geographic coordinates, latitude: 16°43′22″ *S*, longitude: 31°06′52″ *E*, elevation above sea level: 1156) in the Mashonaland Central Province of Zimbabwe. The plant was collected in the vegetative and flowering state in the month of March which coincides with the late summer period in Zimbabwe.

The plant's identity was authenticated by a taxonomist, Mr. Christopher Chapano. Herbarium samples, C1E7, were kept at the National Herbarium and Botanic Garden (Harare, Zimbabwe) and the Department of Biochemistry, University of Zimbabwe. The chemicals which were used in this study were purchased from Sigma-Aldrich Chemical Company (Munich, Germany). The solvents which were used included dichloromethane (DCM), hexane, acetone, ethyl acetate, ethanol, methanol, and dimethyl sulfoxide (DMSO). The standard antibiotics which were used were ampicillin and ciprofloxacin. The media used to culture and grow the bacteria were Luria broth and Luria agar, respectively. Dimethylsulfoxide (DMSO) was used for dissolving the extract and fractions for assay.

### 2.2. Extract Preparation

The roots of *V. adoensis* were separated from the other parts of the plant. The root samples were thoroughly washed under running tap water and dried in a Labcon orbital incubator (Labotec Company, Cape Town, South Africa) at 50°C. The dried root samples were ground into powder using the pestle and mortar. The sample obtained was sieved to obtain a fine powder. The preparation of plant extracts was done as described by Mozirandi and Mukanganyama [26]. Hexane, dichloromethane, acetone, ethyl acetate, ethanol, methanol, and water were the solvents used to extract phytochemicals from the powdered root samples using the maceration technique [27]. DCM and methanol were combined (1 : 1 ratio by volume) and used as solvents to obtain an extract which was referred to as total extract.

### 2.3. Bacteria and Culture Conditions

*S. aureus* (ATCC 9144) and *P. aeruginosa* (ATCC 27853) used in this study were obtained from the Division of Microbiology, Department of Biological Sciences, University of Botswana. The bacteria cells for use in antibacterial activity determination assays were separately inoculated into Luria broth media in the absence of glucose while the medium for bacteria to be used in biofilm assays was supplemented with 1% glucose. The inoculated bacteria were then incubated overnight at 37°C with shaking at 120 rpm in a Lab-Companion incubator (SI300 Incubated Shaker, JeioTech, Korea). Following incubation, the cultures were centrifuged at 3500 rpm for 4 minutes in a Hettich ROTOFIX 32 centrifuge (Tuttlingen, Germany), and the supernatant was discarded. The pellets were washed twice in phosphate-buffered saline and suspended in fresh media. The cells were standardised according to 0.5 McFarland standards to create inoculum densities of 2 × 10^6^ cfu/ml for use in the antibacterial activity determination assay and bacterial broth cultures of 5 × 10^8^ cfu/ml for biofilm assays [26].

### 2.4. Effect of Extracts on the Growth of Bacteria

The root extracts from *V. adoensis* were tested for antibacterial activity against the nosocomial bacteria *P. aeruginosa* and *S. aureus* using the broth microdilution method following guidelines from the European Committee for Antimicrobial Susceptibility Testing (EUCAST) [28]. A mass of 0.004 g of each of the extracts was dissolved in 1 ml dimethylsulfoxide (DMSO) to make a concentration of 4 mg/ml. The stock solution was used to make a concentration of 100 *µ*g/ml of the extracts, which was used to make serial dilutions of each of the extracts. Concentrations of 100 *µ*g/ml, 50 *µ*g/ml, 25 *µ*g/ml, and 12.5 *µ*g/ml were prepared and 100 *µ*l was separately added to the wells of a 96-well microplate. To each of the wells, 100 *µ*l of 0.5 McFarland standardised bacterial cells was added to give a final concentration of 1 × 10^6^ cfu/ml. Ten two-fold serial dilutions of ciprofloxacin (0–100 *µ*g/ml) were prepared, and 100 *µ*l was added to wells of the plate containing an equal volume of cells. The plates were covered and placed in a plastic container consisting of absorbent paper saturated with sterile water. Preincubation absorbance readings of the plate were measured at 590 nm using a microplate reader (Tecan GENios Pro, Grödig, Austria), and the plate was incubated overnight without shaking at 37°C in a LAB Doctor Mini Incubator (MID SCI, USA). The cell density was also determined after 20 hours of incubation. The wells with Luria broth served as the sterility control and the wells with cells alone served as the negative control [29].

### 2.5. Time-Kill Assay

The rate of kill of *P. aeruginosa* by the ethyl acetate extract was determined using a modified method from Sim et al. [28]. Extracts and bacteria used were prepared as described previously for determination of antibacterial activity [29]. Extract concentrations in the range of 12.5–100 *µ*g/ml were separately added to the wells of a 96-well microplate with an equal volume of the standardised bacterial inoculum. Preincubation absorbance readings of the plate were measured at 590 nm using a microplate reader (Tecan GENios Pro microplate reader, Grödig, Austria). The cell density was then measured at 590 nm at 30-minute intervals for the first two hours, followed by two-hour intervals for 8 hours using a microplate reader (Tecan GENios Pro microplate reader, Grödig, Austria). The profiles of killing and regrowth of bacteria were assessed as a function of both time and extract concentrations.

### 2.6. Determination of Membrane Integrity

#### 2.6.1. Protein Leakage Assay

The Lowry method described by Lowry et al. with some modifications was used to determine the effect of the ethyl acetate extract from *V. adoensis* on the leakage of proteins from *P. aeruginosa *[30]. The *P. aeruginosa* cells were cultured in Luria broth overnight at 37°C with shaking. A volume of 200 *µ*l of the cells, which were grown overnight, were subcultured in 200 ml of Luria broth overnight at 37°C with shaking. The subcultured *P*. *aeruginosa* cells were suspended in 0.9% saline to give an absorbance at 600 nm of 1.5 (OD_600_ = 1.5). The cells were treated with 50 *µ*g/ml, 100 *µ*g/ml, and 200 *µ*g/ml of the ethyl acetate extract. The negative control consisted of cells which were suspended in saline. The positive control consisted of cells which were treated with 1 mg/ml of ampicillin since it acts on the cell wall of bacteria. The samples were incubated at 37°C with shaking for 2 hours in a Lab-Companion incubator (S1300 Incubated Shaker, JeioTech, Hettich, Korea). Following incubation, the samples were centrifuged at 3500 rpm for 4 minutes in a ROTOFIX 32 centrifuge (Tuttlingen, Germany). A volume of 250 *µ*l of the supernatant of each sample was collected and treated with an equal volume of sodium hydroxide. A volume of 2.5 ml of freshly prepared Lowry reagent was immediately added to each of the test tubes which contained the sample which was treated with sodium hydroxide. The mixture was incubated for 10 minutes at room temperature. A volume of 2.5 ml of freshly prepared Folin-Ciocalteau reagent was added, and the contents were thoroughly mixed. The samples were run together with different concentrations of the standard protein, bovine serum albumin. The amount of protein that leaked out of the cells was quantified by measuring the absorbance of the samples at 650 nm using a microplate reader (Tecan GENios Pro, Grödig, Austria).

#### 2.6.2. Nucleic Acid Leakage

*P. aeruginosa* cells were grown overnight in 200 ml of Luria broth and then centrifuged at 3500 rpm for 4 minutes (Hettich ROTOFIX 32 centrifuge, Tuttlingen, Germany). The supernatant was discarded, and the pellet was suspended in 0.9% saline to give an OD_600_ = 1.5. The cells were suspended in 50 *µ*g/ml, 100 *µ*g/ml, and 200 *µ*g/ml of the ethyl acetate extract. The positive control of the experiment was treated with 1% sodium dodecyl sulphate (SDS). The negative control had untreated cells. The 50 ml centrifuge tubes which consisted of the samples were incubated at 37°C for 10 minutes with shaking. A volume of 1 ml of each of the test samples was transferred into a clean centrifuge tube and centrifuged at 1100 rpm for 1 minute. The pellet was washed with 1 ml of 0.9% saline. A volume of 3 ml of 0.9% saline was added to the pellet. A volume of 3 *µ*l of propidium iodide (PI) was added to the pellet dissolved in 3 ml of saline. This was thoroughly mixed. The samples were placed in the dark for 10 minutes. The fluorescence was measured at an excitation wavelength of 544 nm and an emission wavelength of 612 nm using an *ʄ*_max_ microplate spectrofluorometer (Molecular Devices, Sunnyvale, USA) [31].

### 2.7. Effect of Extract on Biofilm Formation

The effects of the ethyl acetate extract on the formation of biofilms of *P. aeruginosa* were evaluated in a 96-well microwell plate according to the method by Stepanovic et al. [32]. The ethyl acetate extract was serially diluted to give a final concentration range from 100 to 0.8 *µ*g/ml in the wells. A 100 *µ*l volume of numerically standardised inoculum was dispensed into each of the 6 wells of the 96-well plates (Greiner, Sigma-Aldrich) containing an equal volume of variable concentrations of the extract. Some wells of the plate contained 200 *µ*l of media and served as sterility control. Wells that had media and extract were also included to correct background staining. In some wells of the plate, each bacterial strain (200 *µ*l) was also inoculated in the absence of a plant extract and was considered as a positive control for biofilm formation. The plate was incubated in a nonshaking incubator at 37°C for 72 hours (Labotec Co., Cape Town, South Africa). After incubation, the contents of each well were decanted into a discarded container, and the plate was washed three times with sterile phosphate-buffered saline (pH 7.2) to remove free-floating nonadherent cells. The plates were then inverted and blotted on an absorbent paper towel and allowed to air dry in a sterile environment for 15 minutes [33]. The remaining attached bacteria were heat-fixed by heating the plate at 60°C for 1 hour. The adherent biofilm layers formed in each microwell of the plate were quantified using crystal violet staining. To stain the adherent bacteria, 200 *µ*l of 0.1% crystal violet stain was added to each well, and the plate was covered and incubated at room temperature for 20 minutes. After incubation, the excess stain was rinsed off by decantation, and to remove the unbound dye, the plate was washed three times with distilled water and left to air dry overnight at room temperature. Each of the wells of the plate was filled with 200 *µ*l of 95% ethanol to solubilize the biofilm-associated crystal violet dye from the cells. The optical density (OD) of the stained adherent bacteria was determined by an absorbance microplate reader (Tecan Genios Pro microplate reader, Grödig, Austria) at a wavelength of 590 nm. The percentage inhibition of biofilm formation was calculated using the formula:

(1) $$\begin{array}{c} \%\;biofilm\;inhibition=\left[\frac{\left(AB-EF\right)}{G}\right]\times 100\text{,} \end{array}$$

where AB is the optical density of the stained attached bacteria, EF is the optical density of the stained control cultures without microorganisms, and *G* is the optical density of the bacteria in suspended culture.

#### 2.7.1. Biofilm Detachment Assay

The effect of the ethyl acetate extract on the detachment of *P. aeruginosa* biofilm cells was evaluated using the method of Tan et al. with adaptations [34]. *P. aeruginosa* cells were cultured overnight, and the cells were standardised as described before to create inoculum densities of 5 × 10^8^ cfu/ml. The standardised cells were incubated at 37°C for 72 hours without agitation in the presence and absence of the extract to allow for biofilm formation. The process of allowing the biofilm formation by the cells was performed as described in the inhibition of biofilm formation assay. However, to evaluate the effect of the extract on the detachment of biofilms, the step of decanting well contents was preceded by the addition of 3 *µ*l of sodium dodecyl sulphate (SDS; 10%) to each well, and the mixture was incubated for 30 minutes. After incubation, the suspended culture was then discarded and the plate was washed twice with phosphate-buffered saline.

### 2.8. Effect of Extract on Motility of *P. aeruginosa*

The effect of the ethyl acetate extract on the swarming and swimming motility of *P. aeruginosa* was tested on agar plates containing specialised medium. For the tests, the medium was supplemented with 100 *µ*g/ml final concentration of the ethyl acetate extract*. P. aeruginosa* cells were cultured overnight as previously described and then inoculated onto motility plates in the presence or absence of the extract.

### 2.9. Swarming Assay

Swarming is more than just a form of locomotion and represents a complex adaptation resulting in changes in virulence gene expression and antibiotic resistance [35]. *P. aeruginosa* exhibits swarming motility on semisolid surfaces (0.5 to 0.7% agar). The swarm plates used for evaluating the effect of ethyl acetate extract on swarm motility of *P. aeruginosa* were prepared using 0.5% agar, 0.5% peptone, 0.2% yeast extract, and 1.0% glucose, per 100 ml distilled water [34]. A 250 *µ*l of 200 *µ*g/ml ethyl acetate extract was seeded with 5 ml of the medium and poured immediately on a predried agar plate as an overlay. Two microliters of the *P. aeruginosa* culture were inoculated at the center of the agar once the overlaid agar had solidified, and the plates were incubated at 37°C for 24 hours. The swarming motility was assessed by measuring the circular turbid zones formed by the bacterial cells migrating away from the point of inoculation.

### 2.10. Swimming Assay

Swimming motility is a flagellum-dependent form of movement observed in the Gram-negative bacterium *P. aeruginosa* [36]. Swimming motility was assayed on agar plates according to the method of Bala et al. with some modifications [37]. Swimming plates containing 1% tryptone, 0.5% NaCl, and 0.3% agar were used for the assay. The swim plates were inoculated in the center with 2 *µ*l of *P. aeruginosa* cells in the presence and absence of the extract. The plates were then incubated at 37°C for 24 hours. Swimming halos were visualized manually, and their diameters were measured.

### 2.11. Identification of the Phytochemicals in the Ethyl Acetate Extract Using GC-MS

Since the ethyl acetate extract was shown to have potent antibacterial effects, chemical characterization using GSMS was determined. The analysis of the extract was performed using GC-MS (Agilent Technologies, California, USA). The GC-MS had an AOC-20i auto-sample and a 5 MS (5% diphenyl/95% dimethylpolysiloxane) fused capillary column (30 × 0.25 *µ*m ID × 0.25 *µ*m·df). A TurboMass Gold Detector (Perkin-Elmer) was used for mass determination. The ethyl acetate extract was dissolved in 1 ml of the same solvent. The conditions used during the analysis were an electron ionization system operated in the electron impact mode with ionization energy of 70 eV, helium gas (99.999%) as a carrier gas, at a constant flow rate of 1 ml/min, and the injection volume was 2 *µ*l (split ratio of 10 : 1). The injector temperature was maintained at 250°C, the ion source temperature was 200°C, and the oven temperature was programmed from 110°C for 2 minutes, with an increase of 10°C/minute to 280°C, ending with a 9-minute isothermal at 280°C. Mass spectra were taken at 70 eV. The solvent delay was 0 to 2 minutes. The total GC-MS run time was 36 minutes. The mass detector used was TurboMass Gold-Perkin-Elmer. Interpretation of Mass-Spectrum GC-MS was conducted using the database of the National Institute of Standard and Technology (NIST). The spectrum of the unknown components was compared with the spectrum of known components stored in the NIST library. The name, molecular weight, and structure of the components of the test materials were ascertained.

### 2.12. Haemolysis Assay

The ability of the ethyl acetate extract to cause haemolysis in sheep erythrocytes was determined as described by Noudeh et al. with modifications [38]. A volume of 50 ml of blood was collected from an adult sheep (University of Zimbabwe Animal House) and immediately added to an equal volume of Alsever's solution. After the blood was centrifuged at 3000 rpm for 10 minutes in a Hettich ROTOFIX 32 centrifuge (Tuttlingen, Germany), the supernatant was discarded and the residue was washed three times with a 20% volume of PBS. The washed cells were diluted four-fold with PBS and 500 *µ*l of the cell suspension was incubated with 500 *µ*l of the test sample extract in phosphate buffer saline (PBS) of pH 7.2 for 90 min at 37°C. After incubation, the tubes were spun in a microcentrifuge (HERMLE Z 232 M-2, Wehingen, Germany) at 3000 rpm for a minute, and the resulting supernatant (200 *µ*l) was added to 3 ml of Drabkin's reagent. The positive control had an uncentrifuged mixture of erythrocyte suspension in PBS added to Drabkin's regent. The negative control was prepared by adding 500 *µ*l of the erythrocyte suspension to an equal volume of buffer and centrifuging at 3000 rpm for 1 minute, and then 200 *µ*l of supernatant from this mixture was added to 3 ml of Drabkin's reagent. Aliquots (200 *µ*l) of the supernatants in Drabkin's reagent were placed on 96-well plates. To determine the amount of haemoglobin released, the absorbance of the samples at 590 nm was read using a Tecan GENios Pro microplate reader (Grödig, Austria). The percentage of haemolysis for each sample was calculated by dividing the sample's absorbance by positive control absorbance and then multiplying by one hundred.

### 2.13. Statistical Analysis

Statistical analyses were carried out using GraphPad Prism version 8.0.1 for Windows (GraphPad software, Inc., California). The numerical values were analysed using ordinary one-way ANOVA, with Dunnett's multiple comparison posttest which compares the significant difference at 95% confidence interval. The asterisks (^*∗∗∗*^) indicate a statistical difference in the different concentrations of the media.

## 3. Results

### 3.1. Effect of Extracts on the Growth of Bacteria

**Figure 1:**
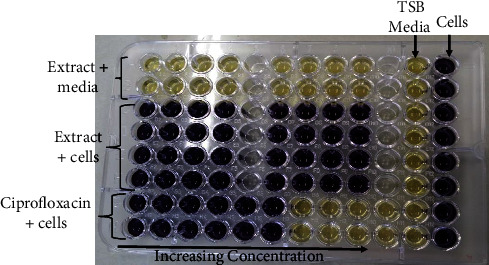
A typical layout of the 96-well plate which was used in determining the effect of acetone extract and ethyl acetate extract on *P. aeruginosa* clinical strain using the MTT assay. Rows A and B consisted of the media and extract which were a negative control. The other negative control was column 11 which consisted of tryptic soy broth (TSB) media alone, hence there was no reduction of the MTT. Column 12 consisted of cells which were not treated and therefore reduced the MTT to purple. The columns (5 and 10) which appeared to be colourless were empty; the acetone extract was present in columns 1–4, while in columns 6–9, ethyl acetate extract was present. The concentration of the extracts and standard antibiotics was increased from left to right. The MIC for the cells treated with the antibiotic ciprofloxacin was 0.125 *µ*g/ml.

The antibacterial activity of each of the crude extracts which were obtained from the roots of *V. adoensis* was determined against both the ATCC and clinical strains of *P. aeruginosa* and *S. aureus* using the microbroth dilution assay. The 3-[4,5-dimethylthiazol-2-yl]-2,5 diphenyl tetrazolium bromide MTT assay was also performed to determine the viable cells after 20 hours of incubation at 37°C. After the addition of MTT, cells were incubated for 2 hours and the production of a purple colour from the yellow colour (initial colour of MTT reagent) indicated the presence of viable cells. The wells in which the media (negative control) only was added remained yellow, indicating that there were no viable cells which could reduce MTT to formazan (Figure 1).

**Figure 2:**
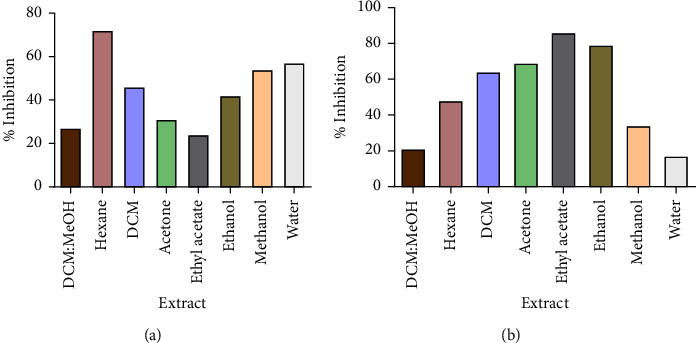
Effects of the different root extracts of *V. adoensis* against *P. aeruginosa*. (a) % inhibition for the clinical strain and (b) % inhibition for the laboratory strain. Concentrations of 100 *µ*g/ml and 1 × 10^6^ cfu/ml of the extract and bacteria were used, respectively. There was no MIC which was obtained for all strains of the bacteria which were screened for antibacterial activity, hence the percentage inhibition of each of the root extracts was calculated.

**Figure 3:**
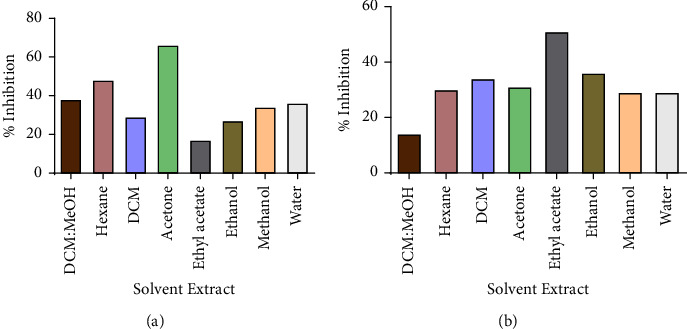
The effect of *V. adoensis* extract on the growth of *S. aureus*: (a) % inhibition for the clinical strain and (b) % inhibition for the ATCC 9144 strain. Concentrations of 100 *µ*g/ml and 1 × 10^6^ cfu/ml of the extract and bacteria were used, respectively.

**Figure 4:**
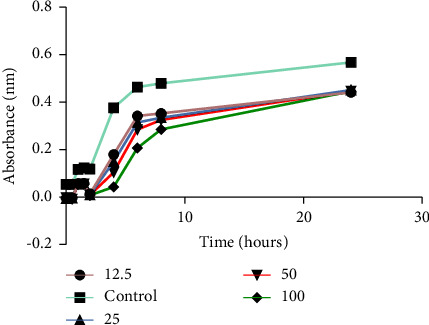
Time-killing activity of the ethyl acetate extract of *P. aeruginosa*.

All extracts had an inhibitory effect on the growth of the test strains as shown in Figures 2 and 3, but there was no concentration that managed to completely inhibit the growth of any of the four bacteria. The ATCC strains of both *P. aeruginosa* and *S. aureus* (Figures 2(b) and 3(b)) were more susceptible to the plant extracts than their clinical strains (Figures 2(a) and 3(a)). The study showed that *P. aeruginosa* was more susceptible to the crude extracts from *V. adoensis* roots compared to *S. aureus*. The ethyl acetate extract was the most potent inhibitor of the growth of the ATCC strain of *P. aeruginosa by* 86% as shown in (Figure 2(b)). Results from the time-kill assay during the 24-hour incubation period with the most potent extract (ethyl acetate) showed a significant reduction in *P. aeruginosa* bacterial population in the presence of the tested concentrations of the extract as shown in Figure 4. There was a reduced number of viable cells present in the cells exposed to the different concentrations of the *V. adoensis* root ethyl acetate extract relative to those not exposed to the extract. However, there was no concentration which managed to result in the death of the bacterial cell.

### 3.2. Protein Leakage Assay

**Figure 5:**
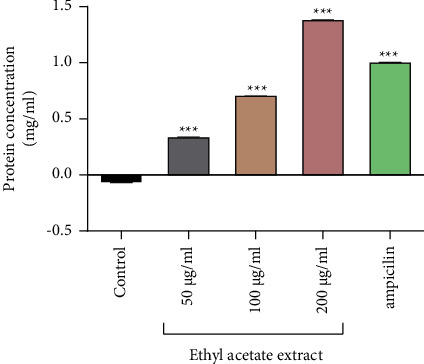
The amount of protein leaked out of the cells in the presence of different concentrations of ethyl acetate extract. Ampicillin (1 mg/ml) was the positive control. The assay was performed in quadruplicate. The significance test was conducted by comparing the samples to the untreated cells using Dunnett's multiple comparison test. ^*∗∗∗*^*P* < 0.0001.

The Lowry method [30] was used in the quantification of the proteins (by measuring the absorbance at 650 nm) which leaked out of the *P. aeruginosa*, as a result of being exposed to the crude ethyl acetate extract. There was a concentration-dependent effect of protein leakage by the ethyl acetate extract on *P. aeruginosa*. A concentration of 200 *µ*g/ml of the ethyl acetate caused the greatest protein leakage from *P. aeruginosa* cells of 1.4 mg/ml (Figure 5).

### 3.3. Nucleic Acid Leakage

**Figure 6:**
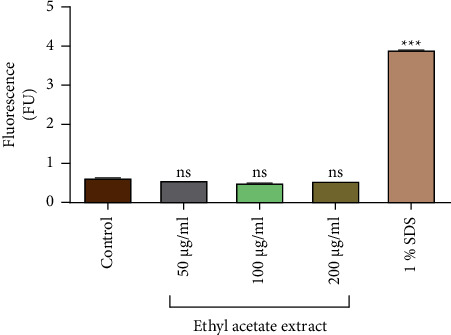
The amount of fluorescence of propidium iodide (PI) measured at 544 nm and an emission wavelength of 612 nm, for determining the effect of different concentrations of the crude ethyl acetate extract of *V. adoensis* on the leakage of nucleic acid from *P. aeruginosa.* The nucleic acid leaks as a result of the disruption of the membrane integrity. 1% SDS was the positive control. The assay was performed in quadruplicate. The significance test was conducted by comparing the samples to the untreated cells using Dunnett's multiple comparison test. ^*∗∗∗*^*P* < 0.0001. NS indicates a lack of significant difference in comparison with the negative control.

The fluorescence of the propidium iodide (PI) was measured to determine the amount of nucleic acid which leaked due to exposure of the *P. aeruginosa* cells to different concentrations of ethyl acetate extract. The ethyl acetate extract did not cause significant leakage of nucleic acid from *P. aeruginosa* (Figure 6). A concentration of 1% of SDS which was used as the positive control caused a significant leakage of nucleic acid, which was shown by the high PI fluorescence of 3.9225 F/unit. At 50 *µ*g/ml of the ethyl acetate, the PI fluorescence was 0.585 F/unit; at 100 *µ*g/ml of the ethyl acetate extract, the PI fluorescence was 0.570; and at 200 *µ*g/ml of the ethyl acetate, the PI fluorescence was 0.560 F/unit. The results were compared with the results from the negative control which consisted of untreated cells (cells only).

### 3.4. Effect of Extracts on Biofilm Formation

**Figure 7:**
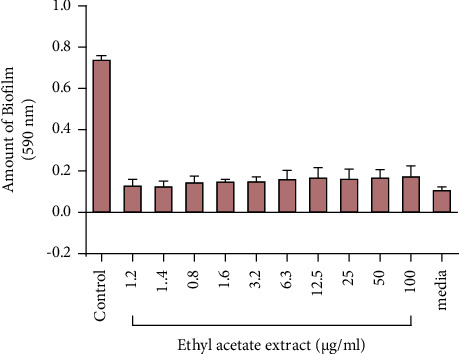
The effect of the ethyl acetate extract on biofilm formation. *P. aeruginosa* (1.6 × 10^6^ cfu/ml) was incubated with different concentrations of the extract for 72 hrs at 37°C, and the resulting biofilm mass was stained using crystal violet staining and quantified spectrophotometrically at 590 nm. The error bars indicate the standard deviation from the mean (*n* = 4). Dunnett's multiple comparison test was used to compare columns by using GraphPad Prism. TSB means tryptic soy broth.

The effect on the formation of biofilm by *P. aeruginosa* was studied after 72 hours of exposure of the test strains to different concentrations of *V. adoensis* extract. The results show that all tested concentrations of the extract completely inhibited the formation of biofilm by *P. aeruginosa* (Figure 7).

### 3.5. Biofilm Detachment Assay

**Figure 8:**
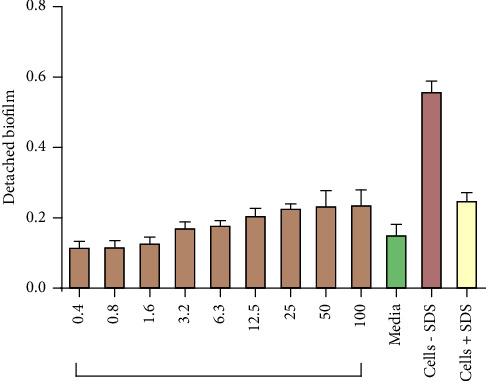
Effect of *V. adoensis* on the detachment of biofilm. *P. aeruginosa* (1.6 × 10^6^ cfu/ml) was incubated with different concentrations of the extract for 72 hrs at 37°C, and the resulting biofilm mass was exposed to the surfactant SDS after which it was washed and the biofilm mass was stained using crystal violet staining and quantified spectrophotometrically at 590 nm.

To evaluate the effect of surfactant on the detachment of biofilm cells, we quantified the amount of biofilm after treatment with SDS in biofilms formed in the presence and absence of different concentrations of *V. adoensis* ethyl acetate extract. SDS detached *P. aeruginosa* biofilm cells from both treated and untreated biofilms as these have lower cell density relative to cells not exposed to the surfactant (Figure 8). The results of the study showed that there was no statistically significant difference in SDS detached cells from untreated biofilms of *P. aeruginosa* and detached cells from cells exposed to the ethyl acetate extract (Figure 8).

### 3.6. Effect of the Extract on Swimming Motility

**Figure 9:**
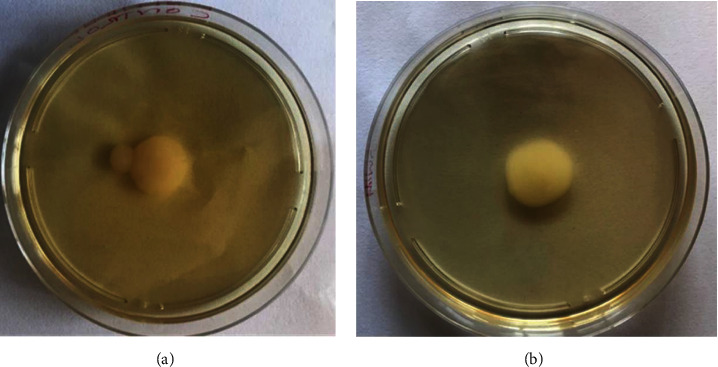
Effect of *V. adoensis* on swimming motility of *P. aeruginosa*: (a) *P. aeruginosa* without extract. (b) *Pseudomonas aeruginosa* with *V. adoensis* extract.

Biofilm formation starts with bacteria swimming towards the surface. It was tested if *V. adoensis* extracts altered the motility of *P. aeruginosa* in plates containing specialised medium, where bacteria can swim, forming a halo from the point of inoculation. Halo size indicates the efficiency of the colony in producing particular enzymes. The results showed that the extract did not affect the swimming motility of *P. aeruginosa.* Cells grown without the extract had a swimming halo diameter of 13 mm (Figure 9(a)), and this was similar to the diameter of the cells with the extract (Figure 9(b)).

### 3.7. Effect of the Extract on Swarming Motility

**Figure 10:**
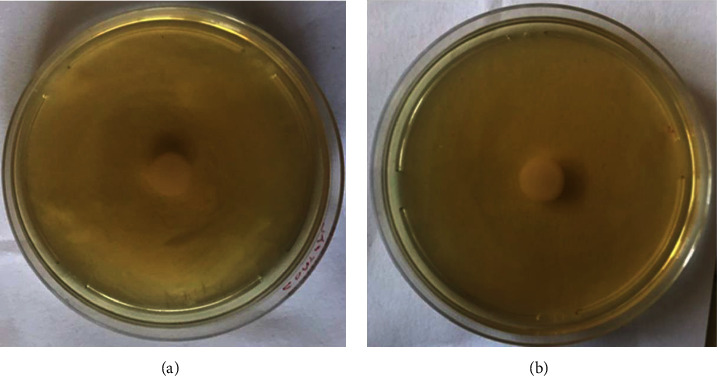
Effect of *V. adoensis* on swarming motility of *P. aeruginosa*: (a) *Pseudomonas aeruginosa* only (without extract). (b) *Pseudomonas aeruginosa* with *V. adoensis* extract.

The swarming motility of *P. aeruginosa* cells grown with and without *V. adoensis* extract was evaluated by growing cells on a swarm agar plate and by measuring the length of dendrites from the center. The dendrite length of cells grown with the extract was 11 mm (Figure 10(b)), and this was similar to the length of cells grown without *V. adoensis* extract (Figure 10(a)). The results of the effect of *V. adoensis* extract on the motility of *P. aeruginosa* cells suggested that the extract had no effect on neither the swimming motility nor swarming motility of the bacteria.

### 3.8. Haemolysis Assay

**Figure 11:**
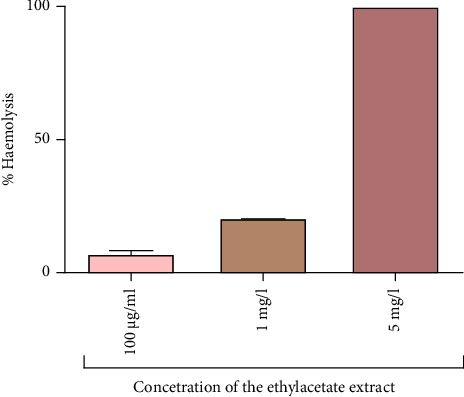
The haemolytic effect of *V. adoensis* extract (100 *µ*g/ml, 1 mg/ml, 5 mg/ml) after 90-minute incubation with sheep erythrocytes at 37°C. Data are represented as mean ± standard deviation (*n* = 4).

Toxicity of the most potent extract was evaluated by determining its ability to haemolyse sheep erythrocytes. The results from the study indicated that the ethyl acetate extract had a concentration-dependent haemolytic effect on sheep erythrocytes. The lowest concentration of extract used, which was 100 *µ*g/ml, did not cause haemolysis of the erythrocytes, while at 1 mg/ml of the extract, 21% haemolysis was observed (Figure 11). The highest concentration of extract (5 mg/ml) exhibited more than 100% haemolytic activity on the erythrocytes.

### 3.9. Identification of Phytochemicals Using the GC-MS

**Figure 12:**
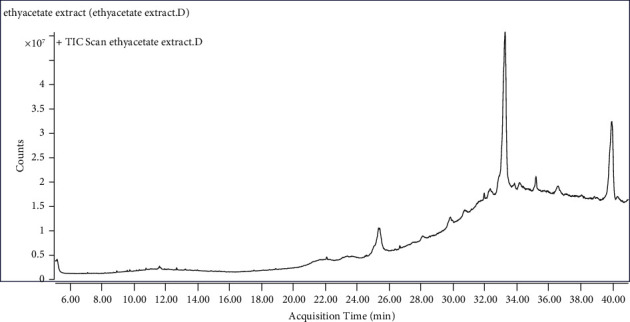
The GC-MS chromatogram was obtained from the ethyl acetate extract from the roots of *V. adoensis.* The spectrum of the unknown component was compared with the spectrum of the known component inherent in the National Institute of Standard Techniques (NIST) library.

**Figure 13:**
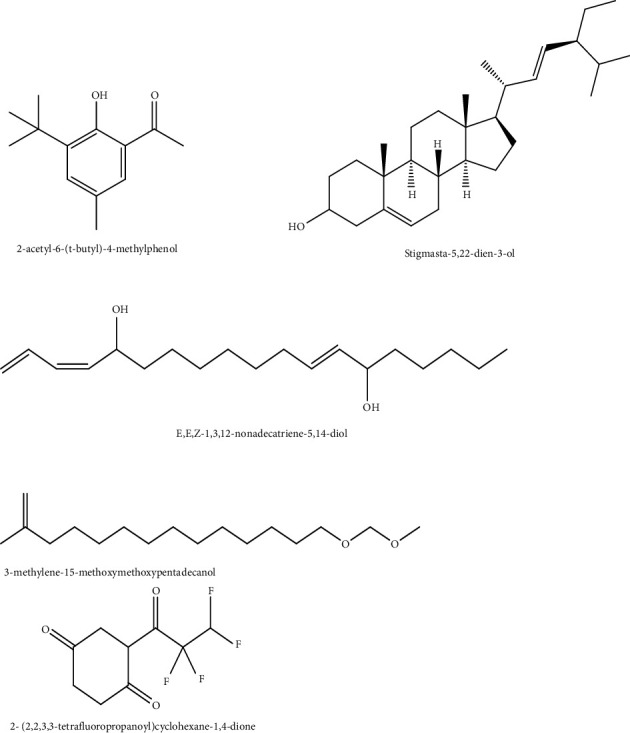
Structure of compounds identified in *V. adoensis* by GC-MS. Interpretation of Mass-Spectrum GC-MS was performed by comparing the spectrum of the unknown components with the spectrum of known components stored in the National Institute of Standard and Technology (NIST library).

GC-MS was used to identify the phytochemicals which were present in the ethyl acetate extract. Interpretation of Mass-Spectrum GC-MS (Figure 12) was performed by comparing the spectrum of the unknown components with the spectrum of known components stored in the National Institute of Standard and Technology (NIST) Mass Spectral Library. The compounds which showed great percentage abundance are shown in Figure 13, and these were considered the bioactive compounds which had the antimicrobial activity observed for the ethyl acetate extract.

## 4. Discussion

### 4.1. Antibacterial Susceptibility

Evaluation of the antibacterial activity of the crude root extracts of *V. adoensis* showed that all the prepared extracts had antibacterial activity against the tested bacteria. These results are in agreement with other studies which have shown that extracts from plants have antibacterial activities [22, 39]. Mozirandi and Mukanganyama evaluated the antibacterial effect of the crude acetone leaf extract of *V. adoensis* against *P. aeruginosa,* and the growth of the bacteria was completely inhibited at 1.6 *µ*g/ml [26]. Compared with the results obtained by Mozirandi and Mukanganyama [26], the leaf extracts of *V. adoensis* had a more antibacterial potency than the crude root extract used in this study. The reason for the differences could be attributed to the fact that the amount and type of phytochemicals which are present in different plant organs vary [40]. Qualitative determination of the phytochemicals which were present in the roots and leaves of *V. adoensis* showed the presence of phenols, saponins, flavonoids, glycosides, and tannins in both the leaves and roots, but terpenoids were only present in the leaves [24]. The terpenoids cause dissolution of the cell wall of bacteria by weakening the membranous tissue, thereby leading to the death of the bacteria [41]. The absence of alkaloids and terpenoids could have led to the relatively reduced antibacterial activity of the root extracts. The ATCC strains of *P. aeruginosa* and *S. aureus* were more susceptible to the root extracts from *V. adoensis*. Root extracts from *V. adoensis* caused low percentage inhibition of growth of the clinical strains of *P. aeruginosa* and *S. aureus*. The clinical strains proved to be resistant to the crude extracts relative to the laboratory strains. The resistance can be attributed to previous exposure of the bacteria to antimicrobial agents which had the same structure with the active components in the crude extracts of *V. adoensis* [42]. *P. aeruginosa* was more susceptible to the root extracts of *V. adoensis* than *S. aureus*. The Gram-positive bacterium *S. aureus* is expected to be more susceptible to the crude extracts in comparison with the Gram-negative bacteria *P. aeruginosa* [43]. *P. aeruginosa* has a more complex cell wall which consists of a thin peptidoglycan layer [44] on the cytoplasmic membrane and an outer membrane composed of phospholipids and lipopolysaccharides [45]. The cell wall of *P. aeruginosa* was expected to restrict the diffusion of phytochemicals which were present in the crude root extracts [46].

### 4.2. Sensitivity of Gram-Positive versus Gram-Negative Bacteria

The plants from *Vernonia* species are proving to have more potency on Gram-negative bacteria than the Gram-positive bacteria [47, 48]. The nonsensitivity of the Gram-positive bacteria can be attributed to the functional groups which were present in the bioactive compounds [41]. Similar findings were found by Lopez where the presence of a carbonyl group in carvone instead of a hydroxyl group in position 3 of carveol was responsible for the susceptibility of the Gram-negative bacteria *Escherichia coli* (*E. coli*) in comparison with the Gram-positive bacteria *S. aureus* [44]. The differences in the shape of the bacterial cells could have also led to differences in the susceptibility of the different strains of the bacteria to the crude extracts. The rod-shaped bacteria are more susceptible to fatty acids in comparison with the cocci bacteria [49]. The rate of kill as well as the mechanism of antibacterial action of the most potent extract was evaluated against the most potent bacteria. Results from the time-kill assay show that there was no concentration which caused the death of bacterial cells, but the used concentration managed to reduce the number of viable cells during the 24-hour study period. The ethyl acetate extract caused significant disruption of the membrane integrity of *P. aeruginosa* resulting in leakage of proteins from the bacterial cells. The negative control which consisted of the cells alone did not cause any protein leakage. The plant phytochemicals such as phenolic compounds and flavonoids could have been attributed to the disruption of the membrane integrity, leading to leakage of the proteins [47]. Studies have shown that tannins and phytochemicals which have phenolic groups as their major constituents are known to prevent the growth of bacteria by disrupting their membrane [46]. The phenolic compounds which were present in the ethyl acetate extract could have interacted with the membrane of *P. aeruginosa,* leading to interference with the growth of the bacteria. In this study, there was no leakage of nucleic acids, since the fluorescence was not significantly different from the untreated cells of *P. aeruginosa*. The bioactive components which were responsible for protein leakage could have interacted with the cell membrane in a mechanism which allowed the leakage of proteins only.

### 4.3. Chemical Characterization of the Ethyl Acetate Extract by GC-MS

When the gas chromatography mass spectrometer (GC-MS) analyses were performed on the ethyl acetate extract, the bioactive compounds which were identified in large quantities were 3-methylene-15-methoxy pentadecanol (fatty alcohol), 2-acetyl-6-(t-butyl)-4-methylphenol (phenolic compound), 2-(2,2,3,3-tetrafluoropropanoyl) cyclohexane-1,4-dione (diketone derivative), E,E,Z-1,3,12-nonadecatriene-5,14-diol (diol), and stigmasta-5,22-dien-3-ol (steroid). The compounds which were identified using the GC-MS were responsible for the antibacterial activity of the ethyl acetate extract and the leakage of the proteins from the bacteria.

The fatty alcohol which was identified using the GC-MS was 3-methylene-15-methoxy pentadecanol. The fatty alcohol could have led to the disruption of the membrane of *P. aeruginosa* leading to the leakage of the proteins. Fatty alcohols are known to disrupt the outer membrane of Gram-negative bacteria through the release of lipopolysaccharides [44]. The fatty alcohol 3-methylene-15-methoxy pentadecanol could have been responsible for the susceptibility of *P. aeruginosa*. The fatty alcohol interacted with the outer membrane of *P. aeruginosa,* hence leading to more susceptibility of *P. aeruginosa* in comparison with *S. aureus*. The absence of the outer membrane in *S. aureus* could have attributed to the low activity of the bioactive compound in *S. aureus.* The phenolic compounds cause inhibition of cell wall synthesis by forming irreversible complexes with proline-rich proteins [9, 50]. The phenolic compound 2-acetyl-6-(t-butyl)-4-methylphenol, which was present in the ethyl acetate extract, could have attributed to the inhibition of growth of *P. aeruginosa* through inhibition of the cell wall synthesis. The isolation of the compound might lead to an enhanced antibacterial activity against both the Gram-negative and Gram-positive bacteria.

The diol which was present in the ethyl acetate, E,E,Z-1,3,12-nonadecatriene-5,14-diol has been found to have antibacterial activity against different types of bacteria [45, 51]. The mechanisms of action of diol have not yet been elucidated [52]. There is a need to determine the mechanism of action of diol against the bacteria, as this may assist in coming up with new antimicrobial agents that may overcome the resistant mechanisms expressed by the bacteria. The antibacterial activity of the ethyl acetate extract may also have been due to the presence of the steroid compound stigmasta-5,22-dien-ol. Stigmata-5,22-dien-ol has been found to be a strong antioxidant having antibacterial activity against multidrug-resistant mycobacteria [41, 49, 51]. The stigmata-5,22-dien-ol could have been responsible for the leakage of proteins of *P. aeruginosa* cells. Steroids are known to cause growth inhibition of bacteria through the interaction with the membrane [53]. A diketone derivative, l, 2-(2,2,3,3-tetrafluoropropanoyl) cyclohexane-1,4-dione was detected in the ethyl acetate extract. The presence of the carbonyl group in the bioactive compound could have led to the antibacterial activity of the l, 2-(2,2,3,3-tetrafluoropropanoyl) cyclohexane-1,4-dione [44]. The ketone derivative interacts with the membrane of the bacteria, thereby causing leakage of the proteins from the bacteria [54]. Bioactive compounds which consist of carbonyl groups have been found to interact with the outer membrane of Gram-negative bacteria, thereby leading to cell lysis [53]. The steroid, fatty acids, ketone derivatives, and phenolic compounds which were present in the ethyl acetate extract caused inhibition of the growth of *P. aeruginosa* through protein leakage. The phytochemicals did not interact with the DNA of the bacteria, hence there were no nucleic acids which leaked out of the bacteria. The compounds which were found to be present in the ethyl acetate extract could be responsible for the antibiofilm activity demonstrated by the crude extract against *P. aeruginosa*. All tested concentrations of the extract used in this study managed to completely inhibit the formation of biofilms *of P. aeruginosa*. The results from the study are in agreement with other studies which have shown plant extracts to be potent biofilm inhibitors [55, 56]. The ability of the extract to completely inhibit biofilm formation makes *V. adoensis* extract a good candidate to help fight biofilm-related infections.

### 4.4. Effects of Extract on Biofilm Formation

The formation of biofilms facilitates the survival of disease-causing pathogens in hostile environmental conditions. Therefore, preventing the pathogen's transition from the planktonic state to the biofilm growth mode is the most important step for combating biofilm-associated pathogens, as the ability of pathogens to resist antibiotics is significantly enhanced 10 to 1000 times once they form biofilms [13, 57]. Clinically, biofilm inhibitors can be used directly to reduce virulence factors from infectious bacteria [58] or to treat biofilm along with conventional antibiotic [59]. Although the extract did not significantly enhance the detachment efficiency of the surfactant SDS, an increase in the detached biofilm was observed with increasing extract concentration. This may imply that higher concentration of *V. adoensis* may enhance the detachment efficiency of SDS. This feature is important in clinical settings where a biofilm is a cause of hazard and increases the cost of treatment for microbial infections. Many acute infections have been shown to be caused by bacteria biofilms adherent to the surface of some medical devices. The introduction of an agent that weakens the biofilm will make it easier for the biofilm attached to surfaces to be easily removed. This can be followed by subsequent biocidal inactivation of the detached biomass [60, 61].

### 4.5. Effects of Extract on Motility of *P. aeruginosa*

*P. aeruginosa* is known to exhibit movement on surfaces by swimming, swarming, and twitching motility. Similar to other motile bacteria, the motility of this bacterium has been shown to be associated with virulence and resistance to antibiotics [62–64]. In this study, we investigated the effects of *V. adoensis* root extract on *P. aeruginosa* swimming and swarming motilities and found that the extract had no effect on the motility of the bacterium. However, there are studies which have shown that plant extracts can inhibit the motility of *P. aeruginosa* [65]. Antibiofilm agents may influence biofilm formation by damaging microbial membrane structures, inhibiting peptidoglycan synthesis and/or modulating quorum sensing [66]. The mechanism of antibiofilm activity of the extract against *P. aeruginosa* remains to be elucidated.

### 4.6. Effects of the Ethyl Acetate Extract on the Membrane Integrity

This study also evaluated the membrane toxicity of the most potent extract. Any agents which have the ability to destroy the erythrocyte membrane can have similar effects on other cell membranes. It was, therefore, important to evaluate erythrocyte membrane stability to determine the toxicity of the most potent extract used in this study. According to the results of the study, the haemolytic activity of the ethyl acetate extracts increased in a dose-dependent manner. On the basis of Fick's law, the diffusion flux from a membrane is proportional to the concentration difference of both sides. In another study by Mozirandi and Mukanganyama [26], 5 mg/ml of extracts from the leaves of *V. adoensis* where shown to exhibit 12% haemolytic activity on sheep erythrocytes compared to 100% at 5 mg/ml for the root extract. This difference may be due to variability in the type and amount of phytochemicals present in different plant organs [24, 40]. When considering the health of consumers, the use of aqueous extract of *V. adoensis* at concentrations with a low haemolytic effect is preferred in pharmaceutical preparations.

## 5. Conclusions

The root extracts of *V. adoensis* have an inhibitory effect on the growth of *S. aureus* and *P. aeruginosa.* The ethyl acetate extract which was the most potent extract completely inhibits the formation of biofilms in *P. aeruginosa* but does not enhance the detachment of the biofilm. The phytochemicals 3-methylene-15-methoxy pentadecanol (fatty alcohol), 2-acetyl-6-(t-butyl)-4-methylphenol, l, 2-(2,2,3,3-tetrafluoropropanoyl) cyclohexane-1,4-dione, E,E,Z-1,3,12-nonadecatriene-5,14-diol, and stigmasta-5,22-dien-ol are present in the ethyl acetate extract. These phytochemicals may have contributed to the growth inhibition of *P. aeruginosa* through disruption of the membrane integrity of the outer membrane causing the leakage of proteins from the bacterial cells. *V. adoensis* roots have the potential to be used as a template to provide agents with both antibacterial and antibiofilm activities. Compounds with antibiofilm activity can be useful during catheter manufacturing as they can be used in the coating of catheters so that no biofilms are formed when they are inserted into patients. Further work needs to be performed to isolate, purify, and identify the specific compounds in the roots that have the antibiofilm activity as well as antibacterial activity.

## Data Availability

The datasets used and/or analysed during the current study are available from the corresponding author on reasonable request.
